# Primate Visual Perception: Motivated Attention in Naturalistic Scenes

**DOI:** 10.3389/fpsyg.2017.00226

**Published:** 2017-02-20

**Authors:** David W. Frank, Dean Sabatinelli

**Affiliations:** ^1^Oklahoma Tobacco Research Center, Stephenson Cancer Center, University of Oklahoma Health Sciences CenterOklahoma City, OK, USA; ^2^BioImaging Research Center, University of GeorgiaAthens, GA, USA; ^3^Division of Neuroscience, University of GeorgiaAthens, GA, USA; ^4^Department of Psychology, University of GeorgiaAthens, GA, USA

**Keywords:** emotion, IPS, LIP, FEF, amygdala

## Abstract

Research has consistently revealed enhanced neural activation corresponding to attended cues coupled with suppression to unattended cues. This attention effect depends both on the spatial features of stimuli and internal task goals. However, a large majority of research supporting this effect involves circumscribed tasks that possess few ecologically relevant characteristics. By comparison, natural scenes have the potential to engage an evolved attention system, which may be characterized by supplemental neural processing and integration compared to mechanisms engaged during reduced experimental paradigms. Here, we describe recent animal and human studies of naturalistic scene viewing to highlight the specific impact of social and affective processes on the neural mechanisms of attention modulation.

## The Fundamental Attention Network

Attention to the surrounding environment allows us to achieve our internally directed goals. Neuronal activation within early visual regions, such as the inferotemporal cortex (IT) and V4, corresponding to attended stimuli is often enhanced while neuronal activity in these areas corresponding to distracting information is suppressed, in part through the influence of regions such as the frontal eye fields (FEFs) and inferior parietal sulci (IPS; Kastner and Ungerleider, 2000; Baluch and Itti, 2011; Carrasco, 2011; Chelazzi et al., 2011). Classically, perceptually salient or unexpected stimuli can involuntarily draw attention in an exogenous, “bottom–up” (BU) fashion (Yantis and Jonides, 1990; Theeuwes, 1992, 2004). In contrast, “top–down” (TD), attention reflects how we voluntarily select items in the environment that merit re-orienting (Posner, 1980; Connor et al., 2004). These two processes may be characterized by different neural mechanisms using networks that ultimately converge and influence one another, and the convergence of BU and TD attention can be described as one, or several, priority maps where stimuli compete for attentional resources (Kusunoki et al., 2000; Bisley and Goldberg, 2010), resulting in one environmental item that draws attention in a “winner take all” fashion. It is important to note that a complex environmental event, as discussed below, may act on both attentional systems and that the activation of these processes is not, necessarily, binary; engagement of endogenous and exogenous attention may lie on a continuum, with specific events assigning different weights to each.

Through learning we establish expectations and rules about the nature of objects within our environment, such that incoming visual information is continuously compared against these expectancies (Summerfield and De Lange, 2014). In this way, we can predictively focus on subsets of the local context and shift attention rapidly should something unexpected occur. Feedforward processing of visual information is monitored via constant feedback from frontal cortex (Miller and Cohen, 2001; Barbas et al., 2011). One such processing stream involves the prefrontal cortex (PFC), which maintains functional connections with FEFs that modulate saccade planning, which in turn projects to the IPS for planning attentional deployment, and continues to primary and secondary visual-processing regions V1 and V2 (Corbetta et al., 2008; Corbetta and Shulman, 2011; Spreng et al., 2013). Additionally, the orbitofrontal cortex (OFC) innervates thalamus and amygdala (Cavada et al., 2000), potentially reflecting affective regulatory functions, which in turn project to ventral visual regions IT, V4, and primary visual cortex for object discrimination (Freese and Amaral, 2005; Tomasi and Volkow, 2011). These regional linkages may enable prior experience to enhance the efficiency and speed of perceptual processing.

Studies of visual attention often involve cue stimuli with little complexity; typically consisting of only a few shapes with solid colors, or motion contrasts on a fixed, blank background (Posner and Cohen, 1984; Wolfe and Horowitz, 2004). While these studies are tractable and extremely valuable in exploring the essential nature of visual attention, they do not resemble the intricacy of naturalistic scenes we encounter in life. To develop a more ecologically representative model of attention processing, it is useful to consider how stimuli that represent realistic daily experiences may affect attentional deployment.

While expectation clearly directs our attentional spotlight in sparse experimental paradigms, we are also interested in how attention circuits differ when processing natural scenes, as contextual cuing incorporates prior experience to expedite visual search (Le-Hoa Võ and Wolfe, 2015). Here, we review recent studies of naturalistic input to the attention process, such as environmental complexity, social stimuli, and affective stimuli. Additionally, we briefly discuss limitations of naturalistic experimental techniques and posit several research problems regarding our understanding of the primate attention network.

## Naturalistic Attention

Humans are exceptionally skilled at rapid detection of other, potentially dangerous, animals in the natural environment. When participants are given prior instruction, scalp event-related potentials (ERPs) can differentiate briefly presented (<25ms) natural scenes containing animals from comparable scenes containing no animals within 150 ms of stimulus onset (Thorpe et al., 1996; Codispoti et al., 2006). Individuals are also able to discriminate peripheral naturalistic images during cognitively demanding tasks. Additionally, this ability does not extend to artificial, but visually salient, stimuli (Li et al., 2002). The efficiency of search concerning natural scenes is, therefore, likely a reflection of our expertise in navigating the world.

Prior exposure with the environment can inform our search strategy to determine where in space to deploy attention. For instance, when searching for a human in an urban context, individuals will first fixate on areas in which humans are typically found; searchers will look for people on a sidewalk before they look on a roof (Ehinger et al., 2009). In the laboratory, individuals will also use prior memory of a novel scene to speed search, engaging both frontoparietal attention mechanisms and the hippocampus (Summerfield et al., 2006). Real-world search is also relatively resistant to the number of distractors. Wolfe et al. (2011) conducted a study in which individuals were asked to find a particular object (e.g., a lamp) located within a natural scene (e.g., a living room) or within a search array (various objects randomly situated on a blank surface). When the target was placed within a natural scene, each additional searchable item added approximately 5 ms to the total search time. However, when targets were placed in an artificial array, each additional distractor added approximately 40 ms to search time. In other words, individuals were much better at disregarding distractors that were logically placed within a natural scene, thus speeding search for the target. Additionally, natural objects placed in locations and orientations typically viewed in the environment reduce cognitive competition compared to items positioned in novel ways (Kaiser et al., 2014). These data demonstrate the considerable impact of context clues in real-world search. Scene context, supported by prior experience, appears to guide TD attention via multiple brain regions, including hippocampus, parahippocampal and occipital place areas, retrosplenial cortex, and IPS (Dilks et al., 2013; Preston et al., 2013; Peelen and Kastner, 2014). In this way, canonical late-stage visual and memory systems are integrated with the attention network, providing regions such as the FEF and IPS with information to significantly modulate visual search.

As we navigate the world, our attentional focus must be continually updated to attain the current goal while inhibiting past goals. In this way, the ventral visual cortex has been shown to be actively suppressed when attending to previous relevant (but now irrelevant) stimuli (Seidl et al., 2012). The ability to rapidly attend to a searched-for object in the environment is influenced by neural preparatory activity from visual regions such as IT. One study has demonstrated that when a person anticipates the presentation of a human in a natural scene, this foreknowledge will enhance IT activation and predict the speed at which the target will be identified. Importantly, this enhanced activity occurs even if no scene is presented, reflecting the preparatory nature of IT in scene perception (Peelen and Kastner, 2011). These data suggest that previous knowledge primes the IT resulting in a more successful search. Additionally, prior knowledge that is no longer useful, and can thus interfere with the task, must be suppressed.

Taken together, the use of naturalistic stimuli in studies of visual search enables a more evolutionarily meaningful examination of attentional processing and its modulation. Attention is also highly efficient when searching quotidian scenes; context derived from experience allows more refined search that directs our focus toward goal-related target areas. The additional information from more realistic stimuli improves visual search and attentional capture by incorporating additional brain regions involved in facial recognition, irrespective of emotion, (e.g., fusiform face area (FFA)), scene representation (e.g., parahippocampal place area, occipital place area, and retrosplenium), and object location (e.g., parietal cortex). Thus, as we move away from highly controlled laboratory tasks and take a more ecologically valid approach, we may then consider the interaction of other neural systems, such as those involved in affective processing, while investigating their effects on attention.

## Emotional Impact on Attention

Although previous experience with contextual cues and episodic memory help guide TD attention, the presence of emotionally evocative cues in a scene has the potential to bias both endogenous and exogenous re-orienting. The attention-grabbing nature of an affectively arousing stimulus is of course a result of natural selection, as rapid orientation to a potentially dangerous (or life sustaining) object will enhance an organism’s likelihood of survival (Lang et al., 1997). Even a weak association of reward can enhance attentional capture by colored singletons in relatively circumscribed laboratory paradigms (Kristjánsson et al., 2010). The communicative value of emotionally expressive faces also modulate attention, as monkeys and humans are faster to attend to threatening images of conspecifics than non-threatening ones (Bethell et al., 2012; Lacreuse et al., 2013; Carretie, 2014). Moreover, averted gaze of conspecifics can be more arousing than viewer-directed gaze, signaling an important environmental stimulus outside of view (Hoffman et al., 2007). Similarly, humans are faster to locate angry face targets, as opposed to happy faces, among neutral stimuli within search arrays of various set sizes (Fox et al., 2000; Eastwood et al., 2001; Tipples et al., 2002). Affective attentional capture has also been illustrated in an emotion-induced attentional blink, where targets are less often detected following an emotional stimulus than a neutral stimulus (Anderson and Phelps, 2001; Keil and Ihssen, 2004; Most et al., 2005; Keil et al., 2006; Arnell et al., 2007). Thus, in situations in which perceptual information is often missed, both emotionally arousing faces and scenes are effective at exogenously capturing attention and are more likely to undergo further visual processing. Taken together, these are but a few illustrations of how affectively arousing stimuli reflexively modulate visual attention.

**FIGURE 1:**
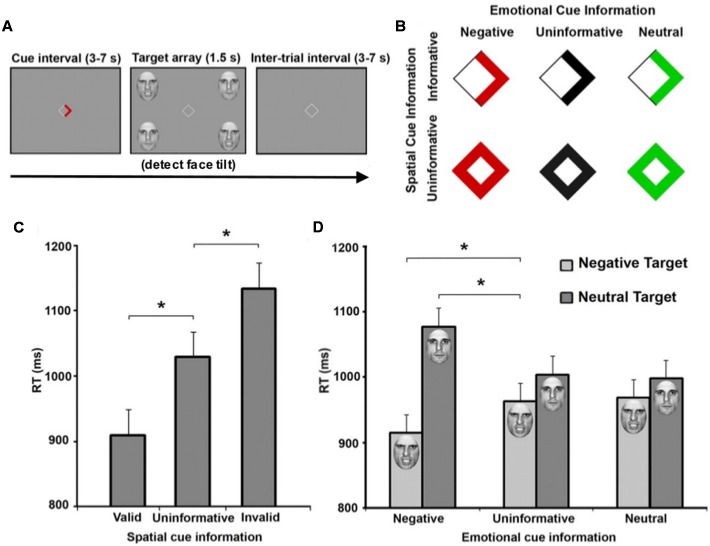
**Emotional and spatial foreknowledge enhances target detection speed**. **(A)** Participants view central cue that may provide emotional information, spatial information, or both about an upcoming target. Participants were instructed to detect the direction of a tilted face among three vertical faces. **(B)** Cues could provide spatial information about the location of an upcoming target, indicated by an arrow, or no information, indicated by a diamond. Cues could also provide emotional information about the face of the upcoming target by the color red, indicating an angry face; the color green, indicating a neutral face; or provide no emotional information, indicated by black symbols. **(C)** Mean response time (RT) of valid (cue-directed attention toward target), uninformative (cue did not direct spatial attention), and invalid trials (cue-directed attention away from target). **(D)** Mean RT of negative, uninformative, and neutral emotional cues when target facial expressions were either negative or neutral. Asterisks indicate statistically significant differences (*p* < 0.05). Adapted with permission from Mohanty et al. (2009).

While emotional attention research has often focused on the ability of an arousing object to redirect attention without explicit instruction, other work has also shown that affective stimuli can modulate goal-directed TD processing. Using a modified Posner paradigm, Mohanty et al. (2009) employed emotionally arousing and non-arousing faces as targets (**Figure 1**). They then manipulated both the spatial location and emotional valence of targets, and both valid spatial cues (arrow direction) and valid emotional cues (arrow color) were displayed to independently speed target detection. Within the emotional cue condition, aversive cues enhanced attention, while both uninformative and neutral cues resulted in no attentional benefit. In fact, uninformative and neutral cues shared similar reaction times. Imaging results indicated enhanced activation in regions including FEF, IPS, and IT in response to spatial cues, while emotional cues additionally evoked amygdala activation. Additive spatial- and emotion-driven effects were found in FEF, IPS, and IT, and functional connectivity between amygdala and IT also increased during emotionally-cued stimuli. These data suggest that the amygdala provides input to an attention network, enhancing our ability to detect affectively arousing targets. Therefore, a set of affective regions, in addition to areas facilitating memory retrieval (e.g., hippocampus), integrate with attention structures commonly identified in controlled experimental paradigms to allow for more efficient and environmentally adaptive behavior.

**FIGURE 2:**
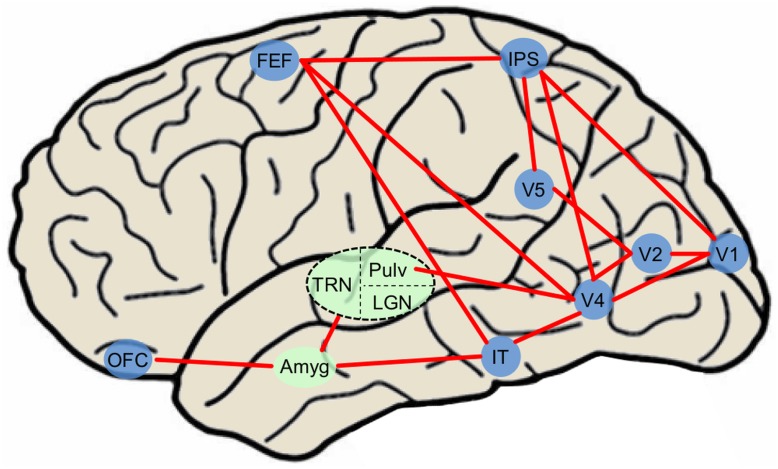
**Simplified diagram of major attention network nodes with the inclusion of affectively modulated regions in a human brain**. Blue nodes denote cortical regions and green nodes denote subcortical nuclei. The dashed oval is subdivided into three thalamic nuclei. Amyg, amygdala; FEF, frontal eye field; IT, inferotemporal cortex; LGN, lateral geniculate nucleus; IPS, intraparietal sulcus; OFC, orbitofrontal cortex; Pulv, pulvinar nucleus; TRN, thalamic reticular nucleus.

Current visual attention network maps (Thompson and Bichot, 2005; Corbetta et al., 2008; Noudoost et al., 2010; Peelen and Kastner, 2014) typically include only canonical visual-processing regions within the dorsal and ventral pathways. In a common attention network model (Pessoa and Adolphs, 2010), the majority of visual stimuli project to primary visual cortex (while some information is sent directly to the superior colliculus). BU processing occurs as visual information progresses throughout the ventral pathway into V2, V4, IT cortex, and synapses on thalamic nuclei such as the medial dorsal nucleus, thalamic reticular nucleus, and pulvinar nucleus that project diffusely throughout the cortex. BU processing also occurs as visual information progresses from V1 along the dorsal pathway to the parietal cortex and FEFs. Meanwhile, PFC exerts TD control over thalamic nuclei and FEF. It is likely that subcortical regions including amygdala modulate BU processing via the current re-entrant model by synapsing onto early ventral visual regions while influencing TD processing through connections with OFC. Due to the ability of emotional stimuli to both exogenously capture and endogenously guide attention, emotionally evocative aspects of stimuli may be incorporated to provide a more accurate picture of an evolved attention system. Structures such as the amygdala have previously been shown to feed into ventral visual cortex creating a re-entrant loop of emotionally enhanced perceptual processing (Amaral and Price, 1984; Freese and Amaral, 2005; Sabatinelli et al., 2009; Sabatinelli et al., 2014), influencing early BU visual attention regions. The amygdala also transacts with regions that influence TD attention such as orbitofrontal and cingulate cortex (Ghashghaei et al., 2007; Pessoa and Adolphs, 2010; Salzman and Fusi, 2010; Saalmann and Kastner, 2011), and may exert control over both TD and BU systems via thalamic connectivity (Pessoa and Adolphs, 2010; Saalmann and Kastner, 2011). Finally, since PFC can attenuate amygdala activity (Rosenkranz and Grace, 2001), TD attention processing originating in OFC may indirectly suppress the effects of emotionally weighted BU attention via amygdala circuitry. Therefore, the interconnected nature of the amygdala allows it to emotionally “tag” stimuli through a variety of neural pathways, and ultimately contributes to the likelihood of orienting to any stimulus in the environment (**Figure 2**). Thus, the inclusion of amygdala and other subcortical structures including regions of the thalamus (Rudrauf et al., 2008; Frank and Sabatinelli, 2014), may serve to refine circuit maps modeling naturalistic visual attention processing. While here we focus particularly on amygdala, this is only one region in a network; other regions likely contribute to affectively modulated attention processing in real-world contexts.

The explicit incorporation of emotion into attention models may also foster greater clinical translation using affective attention tasks to assess emotion-based disorders. For instance, patients with generalized anxiety disorder exhibit stronger emotional attentional blink to threatening stimuli compared to healthy controls (Olatunji et al., 2011). More recently, researchers using emotion-modulated attentional blink tasks have found that soldiers with post-traumatic stress disorder (PTSD) display stronger attentional capture by combat images than do healthy controls or peers not suffering from PTSD (Olatunji et al., 2013). Future attention studies involving the use of real-world stimuli may benefit clinical populations through potential cognitive and neurophysiological attentional redirection techniques, in addition to aiding clinicians with the identification of affective attention biomarkers.

## Conclusion

During naturalistic viewing, attentional deployment to a region of space depends not only on internal goals and the physical impact of light on the retina, but also the context of the scene and experience with the targets involved in the current task. Moreover, the emotional relevance of items in our visual field also impacts attention allocation across exogenous and endogenous pathways. Evolution has resulted in neural mechanisms to discriminate a variety of emotional stimuli, and the guidance of attention by these stimuli likely contributed to human survival; humans can rapidly attend to potential threats or life-sustaining comestibles. Recently, naturalistic attention has been conceptualized as templates to help predict how and where attention will be deployed within our natural world (Peelen and Kastner, 2014). Within this burgeoning area of work, few models explicitly incorporate emotional relevance of targets (Pessoa, 2010). Furthermore, of those studying affective attention, many employ expressive faces, absent of context. It is also true that emotional stimuli can influence both TD and BU attention systems. In fact, some authors have argued that dividing attention into TD and BU divisions is overly simplistic and a third category, namely selection history, should be added (Awh et al., 2012). This point is particularly salient considering affective stimuli can possess both learned and evolved response tendencies. However, it may be the case that the level of TD or BU engagement is dependent on the particular task at hand; context may determine which system is engaged by emotional stimuli.

While the use of naturalistic stimuli will likely open new avenues of research, there are limitations to this methodology. For instance, the perceptual characteristics of scene stimuli, such as image complexity, depth of field, spatial frequency distribution can heavily influence neural activity and act as a confounding variable in an experimental paradigm (Bradley et al., 2007). When addressing the emotional modulation of brain activity, one should recognize that control of hedonic scene content is advantageous (Lang et al., 2008), considering arousal and pleasantness vary across picture categories. As technology advances and virtual reality scene presentation becomes more prevalent, it is possible that more researchers will take advantage of this capability to add another level of ecological validity to their experimental paradigms (Iaria et al., 2008; Nardo et al., 2011). However, this may come at a cost, since a greater number of uncontrolled variables are likely to emerge as an experiment approaches approximating the natural world. It should also be noted that when evaluating emotional attention in non-human primate data, it is often difficult to disentangle attention and emotion, as an animal’s behavior is inherently shaped through reward (Maunsell, 2004). Thus, any conclusions that attempt to differentiate attention and emotion should be taken with caution due to this behavior–reward association, and the inherently intertwined nature of their evolutionary origin.

While social stimuli are powerful cues, as the faces of our peers are effective at communicating dangers and desires, emotion is multi-faceted and there are countless open questions regarding the impact of naturalistic affective stimuli on attention. For example, how do naturalistic affective stimuli differentially modulate BU and TD attention? How do context and individual differences modulate the impact of appetitive and aversive scene processing? What are the limits of TD control on emotional attention? Are variations in these limits associated with disorders of emotion? Multiple studies have demonstrated that a subject’s emotional state influences endogenous attention, speeding reorienting to affective stimuli (Garner et al., 2006; Bar-Haim et al., 2007; Vogt et al., 2011); how does attention-modulation by heightened emotional arousal compare to attention-modulation by declarative knowledge of the upcoming stimulus? These and other questions may be clarified by naturalistic scene research of attentional processing in the real world.

## Author Contributions

DF and DS both provided substantial contribution to the conception and design of the work, drafting the present study, will grant final approval of the version to be published, and agree to be accountable for all aspects of the work.

## Conflict of Interest Statement

The authors declare that the research was conducted in the absence of any commercial or financial relationships that could be construed as a potential conflict of interest.
